# How do countries plan, purchase and use imaging technologies to prevent over-purchase and overuse?

**DOI:** 10.1093/eurpub/ckac129.165

**Published:** 2022-10-25

**Authors:** R Waitzberg

**Affiliations:** 1 Health Systems and Management, Technische Universitat Berlin, Berlin, Germany; 2 Smokler Center for Health Policy Research, Myers-JDC-Brookdale Institute, Jerusalem, Israel

## Abstract

**Background:**

The adoption of new and expensive technologies is one of the main causes of the growing expenditure on health. Regulators are concerned that health care providers purchase (and use) imaging technologies in quantities that exceed the need, increasing expenditure with little value. We review how countries regulate the purchase and use of imaging technologies.

**Methods:**

Qualitative. We collected data using a questionnaire completed by researchers from 17 high-income countries purposefully selected based on variation of policies. We built and compared case studies.

**Results:**

Eleven of the 17 countries analyzed have clear criteria for planning and purchasing imaging technologies. Countries plan different areas, such as supply of specialist care, hospitals by level and type of services, quantity or type of equipment, as well as expenditure on health services and resource allocation. Most countries combine three mechanisms that manage the purchase or use of imaging technologies: (1) seven countries regulate by requiring certificates of need, licenses, or purchase approvals; others regulate by directly limiting the amounts, types and quality of technologies. (2) All countries use financial tools such as activity-based payment, limited and conditional budgets, and caps on income or volume of services. (3) Nine countries centralize purchase by a government agency. The literature provides inconclusive evidence regarding the impact of these mechanisms on expenditure on health and access to imaging services.

**Conclusions:**

Planning the imaging technologies market with clear criteria is essential to avoid abuse. Most countries combine the three mechanisms (regulation, financial tools, centralized purchase). Financial tools are more common and effective. Countries with single payers implement more regulation than countries with multiple, competing, payers. In the later, regulated competition seems to replace regulation. There is a trend of adopting centralized procurement.

**Key messages:**

• Planning the imaging technology market is a precondition to avoid abuse.

• Regulation, financial tools, and centralized purchase can be combined to manage the use of imaging technologies.

